# Floral Biology, Breeding System and Conservation Implications for the Azorean Endemic *Azorina vidalii* (Campanulaceae)

**DOI:** 10.3390/plants14121774

**Published:** 2025-06-10

**Authors:** Rúben M. Correia Rego, Ana Delaunay Caperta, Mónica Moura, Luís Silva, Guilherme Roxo, Roberto Resendes, Maria Olangua-Corral

**Affiliations:** 1 CIBIO, Centro de Investigação em Biodiversidade e Recursos Genéticos, InBIO Laboratório Associado, UNESCO Chair-Land Within Sea: Biodiversity & Sustainability in Atlantic Islands, Faculdade de Ciências e Tecnologias, University of the Azores, 9501-321 Ponta Delgada, Portugal; monica.mt.moura@uac.pt (M.M.); luis.fd.silva@uac.pt (L.S.); guilherme.g.roxo@uac.pt (G.R.); roberto.resendes@uac.pt (R.R.); 2 BIOPOLIS Program in Genomics, Biodiversity and Land Planning, CIBIO de Vairão, 4485-661 Vairão, Portugal; 3 LEAF—Linking Landscape, Environment, Agriculture and Food Research Center, Associate Laboratory TERRA, Instituto Superior de Agronomia, Universidade de Lisboa, Tapada da Ajuda, 1349-017 Lisboa, Portugal; anadelaunay@isa.ulisboa.pt; 4 Banco Germoplasma & Dpto, Biología Reproductiva, Jardín Botánico Canario “Viera y Clavijo”-u.a. CSIC, 35017 Las Palmas de Gran Canaria, Spain; molanguas@gmail.com

**Keywords:** Azores islands, endemic, dichogamy, floral morphology, reproductive biology, self-compatibility

## Abstract

This study investigates the seasonal and floral phenology, breeding strategies, and floral morphology of *Azorina vidalii*, an Azorean endemic Campanulaceae with hermaphroditic, protandrous flowers, dichogamy and secondary pollen presentation. Seasonal phenology was recorded in four field populations and floral phenology in a garden population. Reproductive strategies were assessed via controlled hand pollinations in one field population. Floral morphometrics were analysed using 23 floral and five pollen traits from 121 flowers across fourteen populations throughout the Azores archipelago. Non-parametric and parametric tests, discriminant analysis, and reproductive indices were used to infer answers to this study’s goals. Results showed that temperature and humidity influenced vegetative and reproductive phenophases. The male phase was shorter than the female, likely due to pollen dynamics, and some functional overlap suggested incomplete dichogamy. Geographic variation in floral traits indicated morphological differentiation across subarchipelagos, presumably linked to environmental factors or isolation. Reproductive indices suggested a mixed mating system, partial self–incompatibility and signs of inbreeding depression. Fertilisation was absent without pollinators, and spontaneous selfing was excluded due to an absence of pollen–pistil contact during stigma retraction. These findings contribute to understanding the reproductive biology and morphologic variation in *A. vidalii*. The implications of these findings for the conservation of this insular plant are discussed.

## 1. Introduction

Reproductive studies in plants encompass phenology, floral biology, pollination ecology and breeding strategies, all of which contribute to plant adaptation, evolution, and diversification [1]. Floral polymorphisms, such as dioecy, heterostyly, and flower–pollinator interactions which allow the evolving of pollinator-driven traits, are particularly relevant to outcrossing and fitness [2,3]. However, the transition from outcrossing to selfing is common in plant evolution, especially in island plants (e.g., *Tolpis* Adans., Asteraceae) [4]. While this transition presents some short-term benefits (e.g., reproductive assurance in pollinator-limited environments and increased colonising potential), it has many drawbacks, such as inbreeding depression, possible accumulation of deleterious mutations, genetic diversity losses, reductions in fitness and adaptive potential, and endangering species with extinction [5,6].

The determination of the breeding strategies of plants via artificial pollination experiments in the natural environments highlighted the plasticity of their reproductive systems. In several flowering plants, outcrossing is usually the primary strategy, producing a higher seed set and germination success when compared with selfing [7,8]. However, pollinator limitations, population fragmentation and isolation decrease the natural fruit set in some species [9,10], in which case, reproduction is assured by selfing. This knowledge is important for the establishment of conservation policies, particularly of rare oceanic endemic plants.

The Campanulaceae Juss. family is composed of 95 accepted genera, including over 2300 species [11], most of which are protandrous and exhibit variable degrees of dichogamy [12,13]. In *Campanula* L., both selfing and outcrossing reproduction modes can commonly occur and are influenced by floral traits, pollinator behaviour and availability, and self-incompatibility (SI) systems [12,14,15]. The expression of SI can vary with external temperature, the flower’s age, and the presence of fruits under development, introducing variability into the breeding system of a species across populations [16,17,18].

Much discussion was also employed on the role of pollen-collecting hairs in *Campanula* species [12,14,19]. Manipulative studies showed that the secondary pollen presentation in the stylar brush plays a key role in pollination within *Campanula*, influencing the duration of the male phase and promoting cross-pollination [12,15,20,21]. Selection on a multitude of floral traits [2,22], environmental factors and pollination dynamics are drivers of divergence and adaptation [23,24,25] of phenological and floral morphological traits between species’ populations [26,27,28,29]. In this context, floral geometric morphometrics are a critical tool to comprehend the developmental and evolutionary biology of flowering plants by decomposing key traits such as corolla size, shape and symmetry and their adaptive potential to pollinators and the surrounding environment [30,31]. This is paramount on islands since their geographical isolation and limited pollinator availability often enable island plants to pursue unique evolutionary pathways, promoting geographic differentiation, whose comprehension is essential for conservation [30].

*Azorina vidalii* (H.C.Watson) Feer (Campanulaceae), or *Campanula vidalii* H.C.Watson, as originally named [32], given its clustering with Cape Verde’s *Campanula* species [33], is an endemic, perennial glabrous chamaephyte present in all the islands. It is common in several types of coastal habitats, from sea level to exposed cliffs above 100 m.a.s.l. [34]. This species is threatened by anthropogenic expansion to the coastal areas, being recently targeted in the conservational project Life Vidalia (LIFE17NAT/PT/000510; https://ambiente.azores.gov.pt/lifevidalia/, accessed on 1 May 2025), which performed population reinforcements in the islands of Pico, Faial and São Jorge. Its conservation status was recently reassessed as Endangered, following IUCN criteria [34].

This species has hermaphrodite, protandrous, pendent flowers in a panicle-like inflorescence [35]. Its pollinator network is almost completely composed of insects (Hymenoptera, Lepidoptera and Diptera), with lizard and some wind pollination probably occurring [24,36]. However, interactions between lizards and flowers have remained unexplored in *A. vidalii*. A previous work suggests that this species has strong dichogamy and secondary pollen presentation [24], and preliminary findings point out that selfing and outcrossing may occur, with a slight trend towards outcrossing [37]. However, little is known about its pollination syndromes, and inferences made regarding the existence of SI mechanisms and possible occurrence of selfing within an inflorescence in *A. vidalii* are largely speculative. Some insular species of *Campanula* are self-compatible [17,38], and it was hypothesised that selfing would potentially occur in *A. vidalii* via the stigma’s retraction towards the pollen deposited onto the style [39], though it was never experimentally confirmed. The seeds displayed high germinability in previous in vitro tests [40,41].

Given this information, we hypothesise that *A. vidalii* exhibits a mixed breeding system, with a probable trend towards xenogamy, associated with greater seed production and viability, and some level of self-incompatibility. We also expect some level of floral trait variation across populations due to geographic isolation or environmental pressures, which may influence reproduction and phenology.

This study aims to clarify several aspects of the reproductive biology of *A. vidalii*, which are relevant to its conservation, specifically: (1) flowering phenology in relation to environmental triggers; (2) floral phenology and longevity to access dichogamy and comprehend pollen–pistil interactions; (3) floral morphology and the possible existence of geographic differentiation based on trait variation; (4) breeding system including indirect assessment of self-(in)compatibility; and (5) reproductive success, by assessing seed germination following hand-pollination experiments.

## 2. Materials and Methods

### 2.1. Study Area

**Figure 1:**
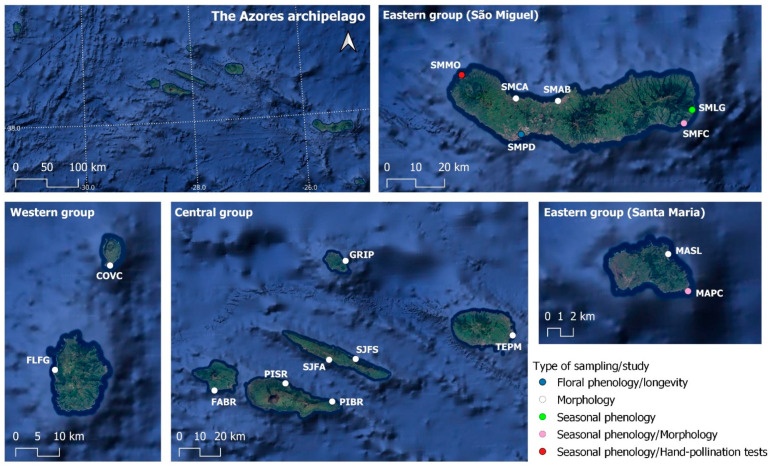
Map of the Azores islands with the sampled populations, categorised by the type of sampling/study performed (Source: Google Satellite).

The Azores is a volcanic oceanic archipelago composed of nine islands, located in the NE North Atlantic Ocean (36°55′–49°43′ N, 24°46′–31°16′ W), covering 650 km between the two farthest islands (Santa Maria and Corvo). The temperate climate is characterised by high humidity, significant precipitation, and low thermal amplitude [34]. Our study was carried out at seventeen coastal populations of *Azorina vidalii*, throughout the nine islands of the Azores. A map with all the sampled locations (Figure 1) was built in QGiS v.3.28.2 [42], and a list with the populations’ information can be found in Supplementary Table S1.

### 2.2. Seasonal Phenology Characterisation in the Field

Our study was carried out in four populations, three in São Miguel (Mosteiros, Lombo Gordo and Fajã do Calhau) and one in Santa Maria Island (Ponta do Castelo). These populations were monitored once a month, but throughout their developmental and reproductive phases (vegetative, flowering and fruiting), the monitoring schedule was shortened to fifteen-day intervals during consecutive years (2022 and 2023). Thirty individuals in each population were selected and marked with plastic labels for monitoring and photographed at each visit.

To determine the predominant phenophase at each visit, a semi-quantitative index was used, following classes of intensity to score vegetative (vegetative, young shoot development) and reproductive (flower buds, open flower, immature and ripe capsules) phenophases in each marked plant: 0 (absence), 1 (when ≤50% of branches are active, low to intermediate intensity), and 2 (>50% of branches active, peak of intensity) [43]. Environmental data (temperature and relative humidity) was collected between 2022 and 2023 at the four monitored populations with the Tinytag^®^ Plus 2 TGP–4500 (Gemini Data Loggers Ltd., West Sussex, UK) data logger.

### 2.3. Flower Phenology Analysis in a Garden Experiment

The flowering phenology was analysed using the species flowering peak date defined as the most recurrent month with maximum flowering intensity throughout the three previous years of observations (2018–2021, see Escobar et al. [44]). The study was conducted in a garden population of *A. vidalii* (population size: 6 individuals), located nearby the University of the Azores, in the centre of Ponta Delgada, during May and June of 2021. Twenty flower buds were tagged at random (four buds per plant) and monitored daily. The stages of flower development and function were studied in buds immediately prior to anthesis, during anthesis, and then in flowers until they reached senescence. Data was collected during the periods 09:00–12:00 and 15:00–18:00 h to determine the duration of the staminate and pistillate phases and floral longevity. Flowers were considered staminate from the time of anthesis to the full expansion of the stigmatic lobes and pistillate from the time of stigmatic lobe expansion to complete senescence. Stigma receptivity was tested on 20 flowers tagged at anthesis from different samples and processed daily until the end of flowering. Flowers were collected and fixed in 70% ethanol, and their receptivity was indirectly evaluated by assessing peroxidase activity using 3% hydrogen peroxide (H_2_O_2_) [45]. Floral longevity was determined by recording the date/time of anthesis and senescence.

### 2.4. Floral Morphometrics

In total, flower samples from fourteen populations of *A. vidalii* were collected throughout the nine Azores Islands. The flowers were sampled from several individuals (minimum of 4, maximum of 27; median: 11.5), according to population size and flowering availability of each field site following Pérez de Paz et al. [46]. Buds and fresh flowers were preserved in plastic containers in a portable cooler with an ice block and fixed in 70% alcohol in the field. Additional flowers and fruits were dried in silica and conserved in paper envelopes. The colour of the corolla was recorded from individuals of each population.

In the laboratory, each mature flower was photographed and subsequently dissected under a magnifying glass (Leica ZOOM 2000, Leica Microsystems GmbH, Wetzlar, Germany) coupled with cold light (Leica CLS 100×, Leica Microsystems GmbH, St. Gallen, Switzerland) [46]. 100 buds, 58 male and 121 female phase flowers were dissected; all floral organs were placed on millimetric paper cards (corolla, calyx and both reproductive structures, gynoecium and androecium) and photographed using a digital camera under a Leica S9i stereomicroscope (Leica Microsystems GmbH, St. Gallen, Switzerland), using LAS Software ver. 4.12.0 image caption software.

A total of 23 floral morphological characters were measured in plants from fourteen populations (Supplementary Table S2). Detailed image analysis and measuring was carried out with Fiji ImageJ software v.1.54f [47]. The images were calibrated to the corresponding metric unit, prior to the measurements, using the scale given by the graph paper cards or an ocular micrometre [46]. For each site/population, morphometric data of the different flower parts, namely calyx, corolla, gynoecium, androecium, pollen and stigmata, were determined.

### 2.5. Pollen Grains and Stigma Observations

From each population, pollen grains of one flower bud were extracted from anthers and allowed to dry any remaining humidity in a stove chamber at 30 °C for 15–30 min. Non-acetolysed pollen grains were then fixed to a microscope slide and stained using a glycerinated hydro-alcoholic solution of basic fuchsin [46] and observed in a light microscope (Nikon Eclipse Ci-L Series, Nikon Corp., Otawara, Japan), coupled to a 12MP Sony Exmor CMOS, E3ISPM Series, digital camera (MicrosCopiaDigital Co., Ltd., Barcelona, Spain).

Additional preparations of pollen grains and stigmas were made using in silico dried samples. These were assembled in metal sample holders, observed and photographed using a Phenom Pro Desktop Scanning Electron Microscope (Thermo Scientific, Eindhoven, Netherlands). In total, 25 pollen grains per population (total *N* = 350) were randomly selected, and spine length, the aperture, equatorial (E) and polar (P) diameters were measured, and the P/E ratio was estimated.

### 2.6. Characterisation of the Breeding System and Evaluation of the Reproductive Success

#### 2.6.1. Pollen/Ovule Ratio Determination

To determine *A. vidalii* breeding strategies, one bud from ten different individuals was randomly sampled in the Mosteiros population (São Miguel Island) and fixed in 70% ethanol. Pollen (P) and ovule (O) amounts were quantified using a modified method of Cruden [48]. The number of pollen grains from each bud was calculated by extracting pollen grains from one anther under a dissecting microscope. Pollen was placed in microscope slides, stained with basic fuchsin and later crushed with a cover slip [46]. After 24–48 h, each sample was observed and photographed, and the amount of pollen grains was counted with Fiji ImageJ. The pollen amount from one anther was later extrapolated for the remaining anthers, giving an estimation of the total pollen amount per bud. The number of ovules per ovary was estimated following the same procedure used for pollen counting, described above. The P/O ratio was calculated by dividing the total pollen count by the total ovule count.

#### 2.6.2. Controlled Manual-Pollination Experiments in a Natural Population

Artificial pollination treatments were performed to study the *A. vidalii* breeding system in the Mosteiros population (São Miguel Island). Each treatment was applied to two flowers per individual (a total of eight flowers per individual) on ten different individuals (population size: 30 plants): (I) control (C): untreated flowers, freely exposed to natural pollination; (II) spontaneous self-pollination (SSP): bagged buds, without manipulation; (III) hand self-pollination (HSP): bagged flowers pollinated using their own pollen; (IV) cross-pollination (X): buds bagged and emasculated before anthesis, and pollinated after anthesis using a mixture of pollen from other plants. To prevent insect visits, all flowers were covered individually with fine polyester mesh bags (pore size ca. 262 mm) before anthesis (bud stage), except flowers from treatment C, which were bagged at the end of the experiment. The bags were collected after the capsules became ripe. For each treatment, the total number of mature seeds and aborted or unfertilised ovules per fruit was counted [49].

Ovaries of ten randomly selected flowers from the same plants were manually dissected to obtain the mean number of ovules per flower. This mean number was then used as a reference value due to the impossibility of counting unfertilised ovules directly in the fruits.

To characterise the breeding system and the levels of self-incompatibility in *A. vidalii*, the following indexes were calculated using the seed (S)/ovule (O) ratios obtained from the pollination experiments: (I) level of autogamy and self-compatibility [50]; (II) selfing rate (s); and (III) level of inbreeding depression (δ) or loss of vigour due to inbreeding, which was calculated using fruit set, seed set, and seed weight of hand-pollinated flowers (wg) and of cross-pollinated flowers (wx): δ = 1–(wg/wx), both according to Charlesworth and Charlesworth [51]; (IV) index of self-incompatibility (ISI), which was calculated by dividing the average number of seeds produced by hand self-pollination to the average number of seeds produced by hand cross-pollination [52] (Supplementary Table S3).

The results were analysed for each separate individual and expressed in percentage since some level of variation is expected to occur between individuals from the same population and then were averaged at the population level.

#### 2.6.3. Seed Viability and Germination

To determine seed viability and germination percentages of seeds produced from either hand self-pollination (HSP) or cross-pollination (X) treatments applied to ten plants in the Mosteiros population (São Miguel Island), germination tests were carried out on fifteen seeds randomly selected from each individual (10 plants, 150 seeds in total per treatment). following the procedure in Menezes [40]. All selected seeds were weighed and measured. Seed samples were immersed in <5% sodium hypochlorite solution (NaOCl) for 5 min. Afterwards, 150 seeds from HSP and 150 seeds of X treatments were incubated separately by individual (20 sets in total) on moist germination paper (Xinxing^®^) in a Petri dish, at 20 °C temperature and with a photoperiod 16 h light/8 h dark cycle [40]. Ten fluorescent lamps delivered light with a photosynthetic photon flux density of 52.5 to 78 µmol·m^−2^·s^−1^. Seeds were observed daily during the first 15 days. An emerged radicle (about 2 mm) was the criterion for germination [53].

### 2.7. Data Analysis

In terms of phenology, the monthly mean intensity of each vegetative and reproductive phenophase, temperature and relative humidity per population was calculated and geographically represented using Microsoft Excel v.2410.

Statistical analysis of flower morphometrics and pollen was carried out in IBM SPSS Statistics^®^ Version 29. To analyse the relationship between *A. vidalii* morphological characters, Principal Component Analysis (PCA) of 23 quantitative morphologic floral traits measured in 121 flowers in the female phase was applied. As a preliminary analysis, the data matrix was reduced to the more relevant variables by successively performing runs of PCA, where the variables that contributed less than 0.70 to the total variance were excluded. We followed Kaiser’s criterium and retained all the components with eigenvalues above 1 [54]. After a satisfactory model was achieved, the principal components retrieved from this analysis were interpreted to assess which variables were correlated in each component and to explain their importance to floral morphology. These components were later included in a multivariate method, namely a discriminant analysis, to assess the occurrence of geographic differentiation based on the variation present in floral morphology, using the islands as the grouping variable. The results were geographically represented in a territorial map, using the first two canonical functions calculated in the discriminant analysis.

Finally, the independent samples *T*-test was employed to study the variance in the floral whorls among flowers in the male or female phase across 19 measured morphometric characters. Anther measurements were not included since they were only measured in male flowers. Levene’s test was used to test the equality of variances and to provide statistical significance.

The occurrence of variation among pollination treatments was analysed using the Kruskal–Wallis test, while the independent samples *T*-test was utilised to detect differences in the S/O ratios obtained among different treatments.

Germination data was analysed with GerminaR software v.2.1.4 [55], following the limits of Ranal and Santana [56] (Supplementary Table S4). This was performed to access the germination times of the seeds resulting from the two types of pollination treatments. To assess significant differences in the germination rates among the two treatments, the independent samples *T*-test was used in IBM SPSS v.29.0.

## 3. Results

### 3.1. Floral Biology and Pollination Syndromes

#### 3.1.1. Seasonal Phenology

**Figure 2:**
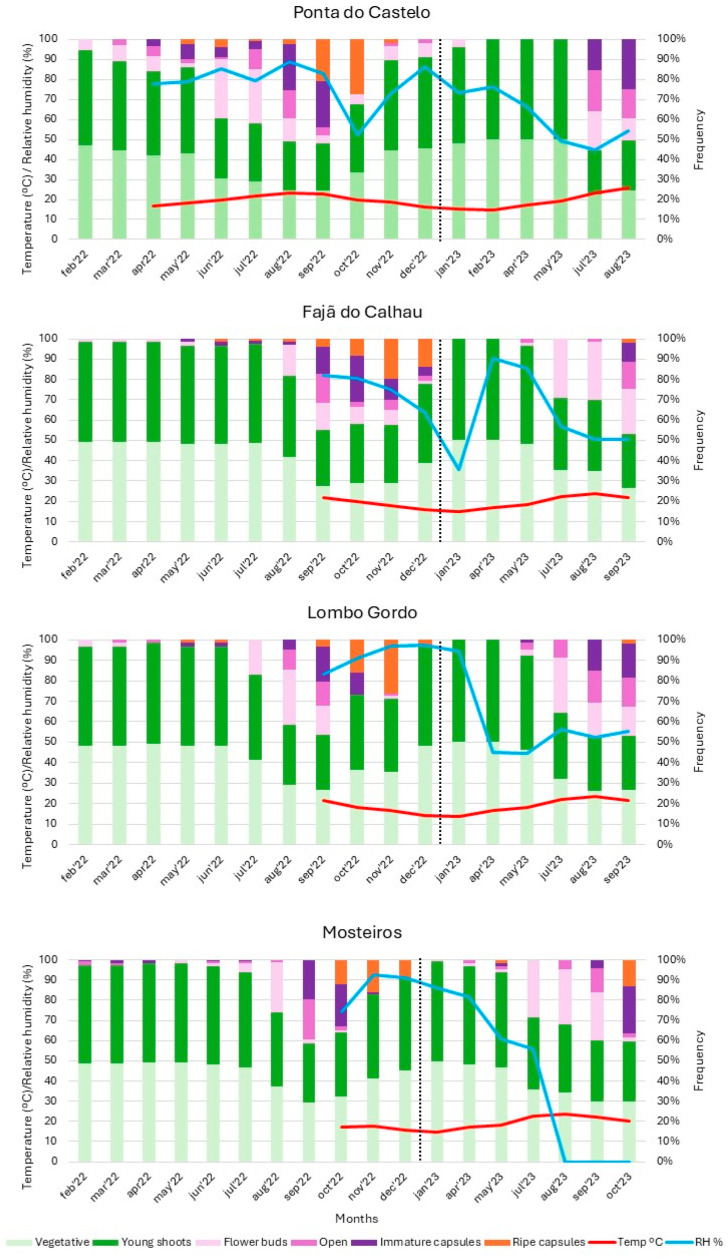
Seasonal phenology of *Azorina vidalii* across four studied populations, respectively, Ponta do Castelo (Santa Maria Island), Fajã do Calhau, Lombo Gordo and Mosteiros (São Miguel Island), with the monthly average temperature (°C) and relative humidity (%).

In general, similar phenological patterns were observed in São Miguel Island populations, showing moderate differences with the phenological pattern of the Ponta do Castelo population in Santa Maria (Figure 2).

*Azorina vidalii* presents vegetative growth throughout the year, reducing its rate during the flowering and fruiting seasons. In all populations, the more intense stage of vegetative growth coincided with a high relative humidity recorded in the previous month.

The data retrieved from the data loggers support that the increase in temperature stimulates inflorescence development between May and July in all the studied populations. The opening of flowers was triggered when the high temperatures settled, during the months of July to September. Whereas in São Miguel Island, flower blooming started in July, with the flowering peak in mid-August (Figure 2), in Santa Maria, it started earlier in June and reached the flowering peak in July. The relative humidity was characterised by drastic variations throughout the day and did not show any visible relation with either flowering or fruiting of this species. Capsule development occurred in the following months, culminating with seed release in October and November. In a few plants, occasional flowering was observed outside the typical flowering period, but the flowers usually abort at the beginning of fruiting.

#### 3.1.2. Floral Phenology

**Figure 3:**
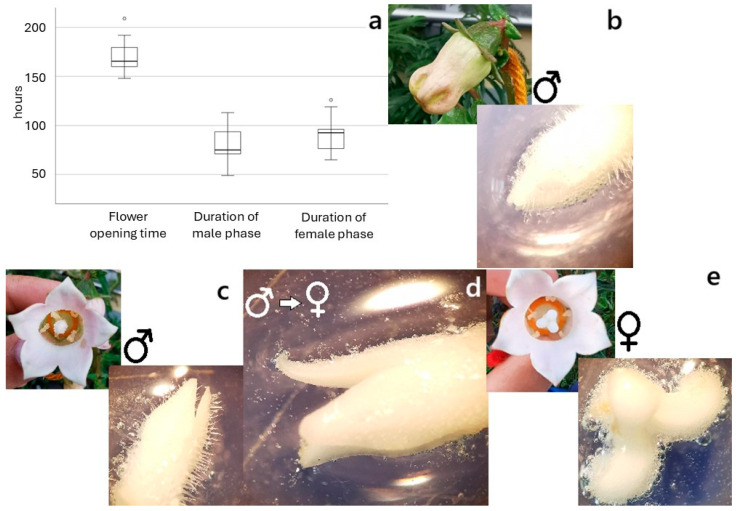
(**a**) Total time of anthesis and duration of functional male and female phases in *Azorina vidalii*. *N* = 20, Anthesis: mean = 169.50 h, range 148–209 h; Male phase: mean = 78.65 h, range 49–113 h; Female phase: mean = 90.70 h, range 65–126 h; Different stages of floral development and stigmatic receptivity throughout the lifespan of the flower of *A. vidalii*: (**b**) stigmatic receptivity in an enclosed bud (**c**); in an open flower in male function; (**d**) during the transition of sex functions; and (**e**) in an open flower in female function. If the stigmata were receptive, bubbles appeared following peroxidase activity assessment, using 3% hydrogen peroxide (H_2_O_2_).

In *A. vidalii*, the order of flower maturation in the inflorescence was acropetal, and different flower stages occurred at the same time in one plant. Flowers had a mean longevity of 169.50 ± 15.93 h, from anthesis until the start of withering, and six stages of flower development were identified (Figure 3):

(I) Bud (bud length: 2.36–2.59 cm): The corolla was enclosed by the calyx, with five bright green fleshy robust sepals;
(II) Bud with visible corolla (bud length: 2.61–2.99 cm): As the corolla elongated, the sepals began to separate, remaining adpressed to the corolla base. The corolla, completely visible, showed a greenish-white or pale-yellow colour (Figure 3). Prior to anthesis, the stamens were rigid, forming a narrow tube around the stigma (filament length (FL): 6.80–7.72 mm; anther length (AL): 10.50–11.01 mm); five closed anthers were slightly in contact with the stigma (style length (SL): 9.70 mm); the three stigmata (~2.60 mm) were immature and tightly closed; and the stamens and pistil extended at the same level, occupying ¾ of the space inside the corolla. The hypanthium diameter reached about 11.30 mm;
(III) Bud, in pre-anthesis (bud length: 3.30–3.62 cm): Corolla colour changes to the usual white or pale pink; the stamens and pistil developed (FL: 10.11–14.00 mm; AL: 11.40–12.84 mm; SL: 10.98–13.90 mm); filaments arched inwards and anthers touched and enclosed the portion of the style with pollen-collecting hairs, and the unexpanded immature stigmata elongated (4.00–5.16 mm). At the end of this stage, the bud started to open slightly, the anthers dehisced introrsely by longitudinal apertures and formed a closed cylinder around the style, releasing pollen through the inside of the anther tube, representing the beginning of the male phase. The hypanthium also attained its maximum size (diameter: 13.81–15.40 mm);
(IV) Flower, in male function: On average, the male function lasted 78.65 ± 15.45 h. The corolla lobes opened, becoming completely recurved at the end of this stage, exposing the sexual organs in the middle of the orange hypanthium. After 24 h of anthesis, the anthers slowly became empty, and complete dehiscence occurred (24–36 h). As the style extended up (14.10–15.40 mm) through the anther tube, sticky pollen was brushed up by stylar hairs (secondary pollen presentation). After shedding pollen, the anthers withered. In this moment, the transition between sex functions started, since the style and stigma elongated to the maximum, and the three stigmata started to open, showing slight receptivity as shown by the peroxidase activity test. Overlapping of functions probably occurred since some pollen was occasionally present in the style and outer part of the stigmas. Dichogamy was apparently incomplete;
(V) Flower, in female function: This phase lasted 90.70 ± 17.50 h. The corolla remained rigid, and the opened stigmata reached the maximum length, recurving completely towards the style, without touching it. Pollen remnants could still be trapped in a few stylar hairs, which did not retract. At this stage the flower was ready for pollination. The stigma was completely receptive, as revealed by the H_2_O_2_ reaction, forming bubbles on the surface of the stigmata (Figure 3). In the following days, the remaining pollen-collecting hairs retracted, and the style became glabrous;
(VI) Withered flower: After three to four days in the pistilate phase, the corolla turned brownish-white and enclosed the pistil.

#### 3.1.3. Lizard Foraging in the Flowers of *Azorina vidalii*

**Figure 4:**
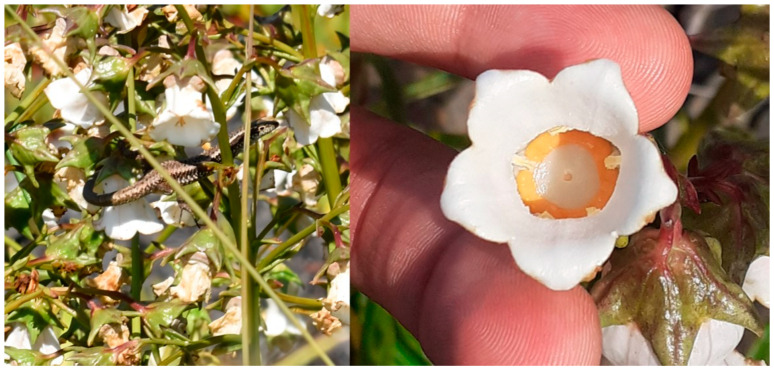
Phenomenon of *Teira dugesii* lizards foraging on the reproductive structures of *A. vidalii* flowers in Ponta do Castelo (Santa Maria Island) (credits: Rúben M. Correia Rego).

We observed that the lizard *Teira dugesii* (Milne-Edwards, 1829) was a heavy forager of this species’ flowers, both in Ponta Castelo (Santa Maria Island) and Areal de Santa Bárbara (São Miguel Island). This is the first report of lizards visiting the flowers on São Miguel Island. We also found many flowers with the stigma and style eaten in these populations, where lizards were seen visiting the flowers (Figure 4).

### 3.2. Flower Morphology Analysis

**Figure 5:**
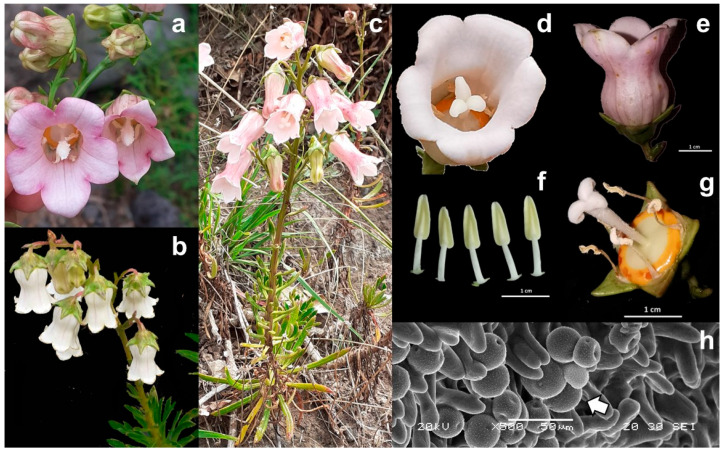
Morphology of *Azorina vidalii*: (**a**–**c**) Examples of floral variation in corolla size and colour in Vila do Corvo (Corvo), Ponta do Castelo (Santa Maria) and Fajã of Santo Cristo (São Jorge Island), respectively; (**d**,**e**) Flower top and lateral views; (**f**) five anthers; (**g**) gynoecium in female phase, with the stigma fully extended; (**h**) pollen grains (white arrow) trapped in the papillous inner surface of the stigmata.

The hermaphrodite flower of *A. vidalii* was actinomorphic, with a pentamerous perianth (Figure 5). The morphometric results are available in the Supplementary Table S5. Calyx was obconic-campanulate, connate, quite thick, with a mean length of 0.59 (range 0.30–0.83) cm, with 5 (4–6) calyx teeth, 1.23 (0.61–1.79) cm in length and 0.63 (0.27–1.00) cm in width. The inferior ovary was fused with the hypanthium and connected to the base of the pedicel. The annulus disc included the nectar disc.

Corolla sympetalous, 2.75 (2.05–3.46) cm in length from the point of insertion until the top of the lobe, 5 (4–6) small lobes, 0.91 (0.53–1.32) cm in length and 0.99 (0.61–1.32) cm in width; campanulate, basally ventricose and constricted in the middle, glabrous outside, hirsute on the inside. It was inserted in the calyx and did not disarticulate during insect visitation. Corolla tube, 1.84 (1.35–2.63) cm in length, the apical perimeter of the corolla tube was 4.34 (2.79–5.91) cm, and the basal perimeter was 4.52 (2.49–6.13) cm. Diameter of aperture 1.29 (0.89–2.06) cm, perimeter 3.84 (2.69–6.47) cm. Maximum diameter from above view is 2.61 (1.48–4.46) cm. The colour of the corolla was pale pink, white, or an intermediate colour between pink and white. Exceptions were found in Fajã do Calhau, where the corollas were white, sometimes intermediate between green–white, while in Corvo, flowers had pink corollas.

The androecium has 5 (4–6) stamens, pale yellow, all equal in size, 1.39 (0.68–1.81) cm in length, on average. Filaments inserted in the hypanthium, above the nectary; white, ⅔ the size of the anther, triangular at the base, from 0.69 (0.27–1.14) cm in length in mature open flowers and 0.66 (0.13–0.99) cm in closed buds, prior to anthesis. Anthers are basifix free, with longitudinal apertures, ranging 0.97 (0.66–1.28) cm in length and 0.29 (0.17–0.45) cm in width, when fully developed and prior to dehiscence.

The gynoecium was unipistillate, with style 1.30 (0.85–2.08) cm in length, covered in pollen-collecting hairs and an orange annular disc at the base. The stigma was trilobate, and after expanding, each stigmata retracted towards the style. When totally opened, each stigma can reach 0.39 (0.26–0.66) cm. SEM images showed that the stigmata had a papillous surface, where pollen grains often got attached, possibly through the action of insects or other pollinating agents. Ovary in an epigynous position; flat-topped, obconic, campanulate in shape, ridged, 0.61 (0.34–0.99) cm in length and diameter of 1.20 (0.76–1.56) cm. Commonly 3 (−4)-locular, composed of many ovules unevenly coupled into lobes. After the capsule became dehiscent after maturation, with erect or slightly nutant valves below the capsule, next to the pedicel, from which flattened seeds were released.

Pollen grains presented isopolar symmetry with spheroidal shape (ratio P/E = 1.01, *N* = 350), tri- or tetra-porate in equatorial view, short aperture and large polar area (Figure 5). The exine was microreticulate, with somewhat uniform spines 0.62 (0.53–0.69) µm, with a polar diameter of 24.97 (21.69–29.82) µm and an equatorial diameter of 24.72 (21.48–29.13) µm.

#### Floral Morphometrics and Detection of Geographic Differentiation

The final model of the Principal Component Analysis used fourteen morphologic floral traits (with communalities > 0.8), composed of six principal components, explaining 89.82% of the variance (Supplementary Table S6). After inspection of the PCA, the first component appeared to be positively influenced by the flower’s size (basal and apical perimeters) and aperture (diameter) and negatively correlated with the filament and stamen lengths. The second component positively correlated the basal perimeter of the calyx, sepal length and anther length and was negatively influenced by the size of the corolla lobes. The third component was positively associated with corolla length and negatively correlated with anther size. For the fourth component, the greatest contributions were from the stamen and filament lengths, which were negatively influenced by floral aperture and diameter. The fifth component was negatively related with calyx characters and positively by anther and stamen lengths, aperture perimeter and corolla tube length. Finally, the sixth component also showed to be negatively influenced by floral aperture and positively correlated with anther size and the corolla tube and lobe lengths.

The discriminant analysis resulted in six canonical functions which explained 100% of the variance (two first canonical functions explained 70.20% of the total variance). The first canonical function was strongly correlated with the third principal component (0.872; Supplementary Table S7) and negatively with the first component (–0.046). While the second canonical function was positively correlated with the first component (0.562) and negatively related with component six (–0.459).

**Figure 6:**
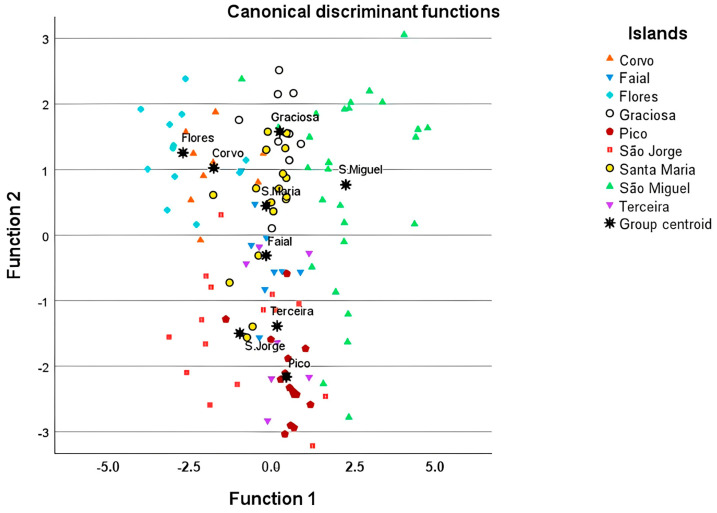
Graphical representation of the geographic differentiation among the nine islands of the Azores, according to the variation present in 23 analysed floral traits measured in 121 flowers of *Azorina vidalii*.

Figure 6 revealed some degree of geographic differentiation, especially at the subarchipelago level, based on floral trait variation. On the negative side of function 1 and the positive side of function 2, the findings revealed samples of the two westernmost islands (Flores and Corvo), whose flowers had shorter corollas but wide apertures and diameters. Furthermore, results showed on the zero of function 1 and negative side of function 2, samples of the islands of the central subarchipelago (Pico, Terceira, Faial, and São Jorge, the latter slightly towards the negative side of function 1), due to the flowers presenting intermediate-sized corollas but smaller diameters and apertures. Moreover, in the zero of function 1 and the positive side of function 2, the samples of Santa Maria and Graciosa Islands are observed, which were only differentiated from the latter by the wider aperture and diameter of the corolla. Lastly, on the positive side of both functions, the group centroid of São Miguel Island, with a few samples appearing scattered towards the negative side of function 2, was revealed. These flowers were characterised by longer corollas with a large diameter and wide aperture.

### 3.3. Breeding System and Reproductive Success Evaluation in Azorina vidalii

#### 3.3.1. Determinations of the Ratio Pollen/Ovule

Pollen and ovule numbers showed considerable intraspecific variation within the Mosteiros population. Pollen production per flower ranged from 144,000 to 1,705,880 grains, with significant differences among all individuals, whereas ovule production per flower ranged from 4468 to 9203. The P/O ratios obtained indicated facultative autogamy for most studied individuals (P/O range: 27.80–162), while only one plant showed to prefer facultative xenogamy as a breeding system (P/O = 293.20). The overall mean value of the P/O ratio was 101.14 ± 77.83, overall pointing to facultative autogamy as the breeding system of the Mosteiros population (São Miguel Island).

#### 3.3.2. Controlled Hand-Pollination Experiments

**Figure 7:**
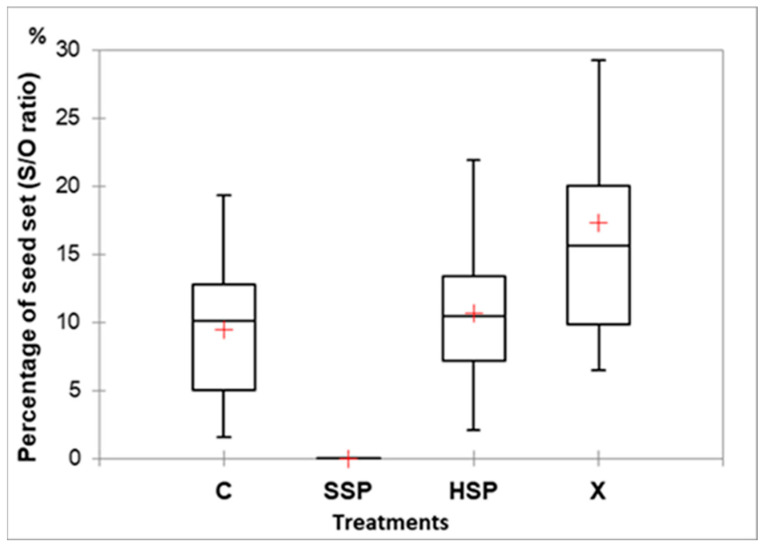
Box-plots with the percentages of seed set, following four pollination treatments, respectively, control (C), spontaneous self-pollination (SSP), hand self-pollination (HSP) and cross-pollination (X), applied to the flowers of ten individuals of *Azorina vidalii*, in Mosteiros (São Miguel Island).

The results of seed set values (S/O ratio in %) for each pollination treatment applied to ten plants in the Mosteiros population (São Miguel Island) are reported in Figure 7. Several bags were lost prior to collection. No seeds were obtained by spontaneous self-pollination (SHP); therefore, this treatment was removed from subsequent analysis.

No differences were detected among the three treatments (C, HSP and X, respectively) following the Kruskal–Wallis test (H = 3.22, df = 2, *p* = 0.20). However, the percentage of seed set differed significantly between treatments (*T*-test, Control: t = 5.27, *p* < 0.001; HSP: t = 6.15, *p* < 0.0002; and X: t = 5.44, *p* = 0.0004; Figure 7). The percentage of seed set from natural pollination (C) was 9.46 ± 5.40%, being, in general, the treatment with the lowest seed production (1.60 ± 19.32%; 100–1169 seeds).

Four individuals had the highest seed production through hand self-pollination (variation between 105 and 1310 seeds), while the remaining six plants produced, via cross-pollination, almost double the seed amount than hand self-pollinated ones (ranging between 379 and 2299 seeds).

The autogamy ratio varied among individuals between facultative xenogamy (three individuals; 0.26–0.45), autogamy (0.63–0.79) and obligate autogamy (0.89–1.21). The population average showed the occurrence of facultative autogamy (0.74).

The autogamy and self-compatibility index retrieved the lowest variation among individuals (only one plant showed to be self-incompatible: 0.021), with 90% of the plants being slightly self-compatible (average value of 0.107, ranging 0.064–0.219).

The index of self-incompatibility implied that 50% of individuals were partially self-incompatible (P-SI), 40% were self-compatible (SC), and only one individual showed to be mostly self-incompatible (0.102, M-SI; Table 1). For the remaining individuals, the values for individual plants ranged from 0.217 to 0.644 (P-SI) and from 1.254 to 2.341 (SC). The population average value exhibited partial self-incompatibility (0.943), being close to self-compatibility.

The inbreeding depression coefficient varied, with four plants showing outcrossing depression and the remaining exhibiting inbreeding depression. The average value for the Mosteiros population revealed a slight tendency for inbreeding depression (0.057).

#### 3.3.3. Seed Germination

As for seed characteristics and germination patterns, the independent samples *T*-test did not retrieve any significant differences across parameters between hand self-pollination and cross-pollination (*p* > 0.05). The weight of the seed sets for the two treatments varied between 0.2 and 0.8 mg (mean 0.49 ± 0.2 mg) in the seeds from self-pollinated flowers and 0.2 and 1.1 mg (mean 0.64 ± 0.3 mg) from crossed ones.

Seeds also varied in length, with the minimum average length ranging from 0.754 mm in cross-pollinated seeds to 0.782 mm in manual self-pollinated seeds, while the average maximum length was around 0.858 mm in seeds from both treatments.

Radicle emergence in both treatments started on the fourth day of the germination trials, with seed germination in both seed sets (83.3% of seeds from hand self-pollinated flowers and 73.3% of seeds from cross-pollinated flowers). After the ten-day experiment, 93.33% of the seeds from manually self-pollinated flowers had germinated (140 seeds), ranging from 86.66% to 100% of germinated seeds among the ten replicates of this treatment. The seeds from cross-pollinated flowers showed a slightly lower percentage of germination (82%, 123 seeds), and the germination percentage among the ten replicates of this treatment varied between 26.6% and 100% (Table 1). Mean germination time (Mgt) was about 4–5 days, with averages of 4.14 for hand self-pollinated seeds and 4.19 for cross-pollinated, and the mean germination rate (Mgr) ranged between 0.22 and 0.25 days^−1^ across replicates from seeds of both treatments.

## 4. Discussion

### 4.1. Influence of Environmental Conditions in the Seasonal Phenology of Azorina vidalii

The Azorean climate is characterised by drastic variations in relative humidity in the summer, becoming higher in the winter months [57]. High humidity might increase *A. vidalii*’s vegetative growth, as observed in other plants [58], slowing down during the reproductive phenophases due to resource allocation [59]. Moreover, the development of inflorescences and the flowering peak appeared synchronised with increasing temperature, as similarly observed in other Campanulaceae [60]. This explains the earlier onset of blooming that occurs on Santa Maria Island, given the higher temperatures and drought found on this island, compared to São Miguel [57]. In this context, climate change can seriously affect the flowering times of coastal endemic plants [61]. As temperature rises, anticipation of *A. vidalii* flowering times may be expected.

Genetic studies regarding flowering time regulation have provided insight about the importance of flowering time diversity in domestication, adaptation and evolution, assuring and maximising plant fitness [62]. For *A. vidalii*, given the dependence on pollinators, the flowering time at summer’s hottest period, coupled with a moderate synchrony of flowering and development of large, many-flowered inflorescences, might be considered an evolutionary adaptation to increase the chances of successful pollination and reproduction by taking advantage of the greater pollinator activity observed during the summer (RMC Rego, personal observation).

### 4.2. Floral Longevity, Dichogamy and Sexual Functions

The flowering peak in six potted *Azorina vidalii* plants differed from that in the natural populations on São Miguel Island, likely reflecting differing abiotic conditions [63]. Based on our observations, the species has long-lived flowers, and the duration of sexual phases shows plasticity due to pollen dynamics [64,65,66], with a shortened male phase due to pollen removal by insects [12,24] and a female phase that can be sometimes twice as long as the male, accelerating stigma development, as found in the genera *Campanula* [20,64,67], *Lobelia* Plum. ex L. [65] and *Centropogon* C. Presl [66]. Such traits should optimise outcrossing efficiency and reduce maintenance costs [68]. However, other traits, such as the early release of pollen (occurring before or at the point of anthesis), signs of early stigma receptivity and an apparent delayed retraction of the stylar hairs, suggest an increased selfing potential and incomplete dichogamy, contrasting with earlier findings [24].

### 4.3. Floral Features and Pollination Syndromes

In this study, we offer an updated floral description of *A. vidalii*, including a detailed floral morphometric study, which hopefully will facilitate future works devoted to taxonomic treatment and reassessment of this species. Additionally, it provides important considerations for this species’ conservation (discussed below).

*Azorina vidalii* has relatively large flowers, unusual for oceanic island floras [69]. Furthermore, floral morphological traits such as their actinomorphic symmetry, wide tubular corolla, campanulate shape and fused petals support the entomophilous syndrome previously suggested [24,36,70], by easing the pollination and foraging by large insects, such as Lepidopterans and larger bees [71,72,73]. The common ancestor of extant Campanulaceae taxa possibly had bilateral floral symmetry and a simpler mechanism of secondary pollen presentation without stylar growth. Improvement of pollen deposition and uptake by pollinators led to evolutionary back-and-fourths between radial and bilateral symmetric floral forms. Ultimately, the reversal to actinomorphic flowers in Campanuloidae was accompanied by the retraction of the stylar hairs, potentially allowing a more successful pollen transfer to pollinators [72,73], given the grains’ sticky consistence (pollenkitt) [74] and presence of spines that adhere to the insects’ bodies [75,76].

Pollination by birds is unlikely for *A. vidalii* due to the lack of observations [24], and the only known pollinating bird in the Azores is the Eurasian blackcap, *Sylvia atricapilla gularis* (Alexander, 1898) which pollinates *Aloe arborescens* Mill. (Asphodelaceae) [36]. The flowers also have traits that suggest anemophily, such as faint scent, abundant pollen, low nectar production, and hairy stigmas [24,77]; however, since they are pendent, such a mechanism will only facilitate selfing [19].

The corolla colour in *A. vidalii* also showed slight variation, from greenish-white to pink; however, this trait appears more related to phylogeny than to pollinator attraction [24], given the absence of specialised pollinators of this species [24,36]. We argue that corolla colour plasticity can be influenced by niche characteristics (substrate, nutrient availability, temperature and humidity) [34] or abiotic factors (e.g., temperature, humidity, solar radiation), which in other species affects pigment production, petal reflectance and colour perception by pollinating insects [78,79].

#### Lizards as Pollinators or Floral Predators?

While in this work, *A. vidalii* pollinators were not studied, we carried out an extensive sampling throughout all the Azores Islands. We discovered that lizard foraging not only occurs in Santa Maria [24] but also in São Miguel Island (Areal de Santa Bárbara). *T. dugesii* was not a main *A. vidalii* pollinator; they are opportunistic floral predators that consume large amounts of nectar and pollen deposited on the style, effectively consuming the female structures. This compromises fertilisation, fruit and seed set production, decreases the pollen load available to effective pollinators, and has direct consequences for plant fitness, population maintenance and conservation, as seen in *Campanulastrum americanum* L. (Small), Campanulaceae [23].

### 4.4. Morphological Variation and Geographical Differentiation

The PCA and the discriminant analysis demonstrated some level of geographic differentiation, even at a preliminary level, due to the variation found in floral aperture and corolla size. These traits have been positively correlated with the size of pollinating insects in some *Campanula* species [80], suggesting a possible diversity of pollinating insects among different islands [24,36]. In a broader context, floral morphological variation in island plants has been attributed to genetic causes (genetic drift, founder effects, adaptive radiation or limited gene flow) [26,27], geographic isolation, pollinator availability, hybridisation, environmental factors and human impacts [29,81].

Our results showed that floral trait variation in *A. vidalii* led to some degree of geographical differentiation at the subarchipelago level (western and central subarchipelagos, except Graciosa Island), agreeing with the hypothesis that adjoining islands have larger flower similarities (central islands) than those from distant islands. We argue that the greater geographic isolation of the westernmost islands is a driver of floral variation, especially in Corvo Island, supported by an intricate relationship between phenotypic variation in floral traits (pronounced shortened corollas) and pollinator fauna, as seen elsewhere [81,82]. In this case, the pollinator network probably shifted from an endemic specialist to a more restricted network composed of a mix of native and introduced insects after human settlement [36].

The centroids of the central islands of Pico, São Jorge, Terceira and Faial appeared to be grouped, and we also found some admixture, with the samples of the islands of Santa Maria, São Miguel and Graciosa appearing somewhat scattered with some samples of the central islands. Some admixture was also found at the molecular level, according to previous phylogenies [33,83]. Both the floral similarities among the central islands and the admixture between more dispersed islands are more likely related to gene flow promoted by seed dispersal, rather than pollination. In the case of this species, there are several vehicles to dispersal, including birds that transport seeds in the gut [84] or on their feet (mud dispersal [85]), anemochory (wind dispersal) and sea dispersal, which would be capable of long and inter-island dispersal of *A. vidalii*’s light-weight seeds, as seen in other archipelagos (e.g., Galápagos [86]). Human-mediated translocations probably occurred since *A. vidalii* is being used as an ornamental plant, and conservation initiatives promoted population reinforcement and translocation [34].

Furthermore, abiotic factors related to the exposure to harsh coastal conditions, salinity, extreme climate, and poor substrates [34] could alter this species’ selective pressures, possibly contributing to fitness and floral morphological variation, as observed in *Calceolaria* L. spp. (Calceolariaceae) [29]. In conclusion, while this is still a preliminary approach, future work integrating more populations for greater sampling would allow a better understanding of the factors that influence morphologic floral trait variation in *A. vidalii* across the islands.

### 4.5. Breeding Systems and Reproductive Success

In this study it was found that *A. vidalii*’s population of Mosteiros (São Miguel Island) have a mixed breeding system, with both autogamy and xenogamy occurring among these plants, as already found across other *Campanula* spp. [15]. Spontaneous self-fertilisation did not occur in *A. vidalii*, since fecundity was not achieved in the insect-excluded bagged flower buds, as observed in other Campanulaceae [19,87,88], ruling out late self-pollination as previously hypothesised [39]. This indicates that pollinator availability is crucial for successful fertilisation in this species.

Despite that, and the presence in *A. vidalii* of traits that would favour cross-pollination, such as large pollen loads and secondary pollen presentation, there was a slight trend towards autogamy in the studied population, found in both the artificial experiments and the P/O ratios, contributing to inbreeding depression. These results contrast with preliminary findings that indicated xenogamy for another *A. vidalii* population of São Miguel Island [37]. As discussed above, this species presents great selfing potential, which combined with an incomplete dichogamy, pollinator dependency and lizard foraging will pose a limitation to the occurrence of natural gene flow through cross-pollination [5]. In this context, our team is developing a population genetic characterisation of *A. vidalii*, which could provide more data about the occurrence of inbreeding depression and patterns of gene flow at a wider range. The level of intraspecific variation noticed in the Mosteiros population is common within individuals and among populations and is likely associated with differing levels of self-(in)compatibility among individuals [89]. In some *Campanula* species, it was determined that the secondary pollen presentation mechanism causes variation in the rates of self- and cross-pollination due to pollen placement dynamics [90].

The findings also revealed that both selfing and outcrossing reproductive modes have led to variable seed sets across treatments and individuals, but as expected, they were often much higher in cross-pollinated fruits [7,8]. However, seeds from both reproduction modes produced similar germination results under optimal conditions of temperature and luminosity, highlighting that reproduction can be successful in this species, regardless of the breeding strategy, and contribute to the maintenance of viable populations.

#### Self-Incompatibility in *A. vidalii* Following Field Experiments

Genetic SI systems in most flowering plants are based on two mechanisms located on the S-locus: one that detects self-pollen and another that rejects it. That way, selfing is prevented, promoting outcrossing and increasing the genetic diversity of the offspring [91]. Although in this study, a direct determination of the *A. vidalli* SI system was not realised, the findings revealed that the studied plants exhibited total to partial self-incompatibility, but still 40% of the studied individuals were slightly self-compatible (SC). While at a smaller scale (only one population studied), our results are somewhat in accordance with those observed for *Campanula rapunculoides* L. (Campanulaceae), with most plants from two natural populations showing total to partial SI and 15% of them exhibiting weak SI [92]. Moreover, it was demonstrated that inbreeding was higher in plants with stronger SI in the partially self-incompatible *C. rapunculoides* [87], similarly to our observations. However, in *A. vidalii*, the plants that exhibited strong SI produced higher outcrossed seed sets, while in *C. rapunculoides* it was the opposite [93], probably due to the action of SI or inbreeding depression [88]. In SI/SC species, with mixed breeding systems, but with a higher trend towards autogamy like *A. vidalii*, the shorter male phase is mostly adaptive, ensuring self-pollen viability when reaching the stigma, but also avoiding complete pollen load depletion from insects prior to the expansion of the stigmatic lobes [12,14,15]. Nevertheless, the evolution of protandry and pollen-collecting hairs under island conditions might have led to a shift from an SI to an SC system, as seen in *Campanula punctata* Lam. (Campanulaceae) [17], since plants that can self-fertilise are more capable colonisers [94].

Expression of SI in *A. vidalii* can possibly be affected by abiotic (e.g., temperature) or biotic factors, like the presence of different phenophases (flowers and developing fruits) or flower age, potentially weakening SI responses, as observed elsewhere [92]. Given the increase in temperature in the summer, which coincides with the plants’ flowering peak, we hypothesise that the rising temperatures cause a less effective response or even breakdown of the plants’ SI system, as found in *Arabidopsis thaliana* (L.) Heynh. (Brassicaceae) or in citrus trees [95,96]. Furthermore, considering that *A. vidalli* is polyploid [97], it is also possible that genome doubling by polyploidisation could weaken, to some extent, the incompatibility barriers by affecting loci that regulate SI systems [96,98]. Ultimately, a genetic approach unveiling the loci that regulate SI and its disrupting factors in *A. vidalii* would be valuable, not only for knowledge of its breeding strategies but also for conservation of this species.

### 4.6. Conservation Remarks

*Azorina vidalii* is threatened by anthropogenic expansion to the coastal areas, invasive species, pollution, habitat fragmentation and extreme weather, leading to habitat destruction, changes in the native plant communities and population depauperation [34]. These threats can pose difficulties to this species’ pollination and lead to geographic isolation. Therefore, the full characterisation of this species’ life cycle, performed here, will be important for conservation planning [99]. The occurrence of inbreeding depression related to greater selfing potential [51] in the Mosteiros population is concerning, given that the same can be occurring in more populations, and an incomplete dichogamy and partial SI do not preclude selfing. To mitigate possible genetic diversity losses, a combination of in situ (e.g., habitat restoration and assisted gene flow) and ex situ (e.g., seed banks) measures will be vital [34].

The detailed floral morphological data contributes to the identification of traits important in plant–pollinator interactions [81]. Findings related to lizard predation and possible negative effects on the plants’ reproduction and fitness are relevant and should be addressed in future conservation efforts. Moreover, quantification of pollinator insects is important and could be assessed in future works. Despite the lack of molecular data about the patterns of population structure and genetic diversity across the species’ geographical range, results from previous phylogenetic studies, namely the occurrence of endemic ribotypes in São Jorge, in the westernmost islands (Flores and Corvo), and shared ribotypes among the central islands [33,83] are also important findings. Moreover, our spatial clustering supports the occurrence of genetic diversity and morphological differentiation within and between *A. vidalii* populations across the islands. This is a fundamental factor for understanding plant trait evolution [29,81]. Ultimately, this study will allow more effective planning and execution of future conservation initiatives.

## 5. Conclusions

This study provides insight on the reproductive biology of the endangered Azorean endemic *Azorina vidalii* and the most comprehensive floral morphology analysis to date, covering 14 populations across all nine Azores Islands, surpassing a previous work [37]. We confirmed that environmental conditions and flower polymorphisms significantly influence phenology and floral longevity of *A. vidalii*. As hypothesised, floral trait variation contributes to some degree of geographic differentiation, especially between subarchipelagos, suggesting an adaptive response to local abiotic pressures and pollinator availability. Artificial pollination experiments revealed a mixed breeding system for *A. vidalii*, which is pollinator-dependent. An incomplete dichogamy and partial self-incompatibility enable some level of autogamy and inbreeding in the studied population, despite the presence of mechanisms that would promote outcrossing. Insular conditions might promote plasticity in floral traits and breeding strategies of *A. vidalii*, but pollination dynamics, lizard foraging and anthropogenic threats can seriously affect outcrossing rates. This work concludes that it is imperative to implement conservation measures that preserve the morphologic variation and genetic diversity across populations by complementing these data with population genetics.

## Figures and Tables

**Table 1:** Mean values of seed germination parameters calculated with GerminR for *Azorina vidalii*, based on 10 replicates, across two treatments: hand self-pollination (HSP) and cross-pollination (X) in the Mosteiros population (São Miguel Island). M: mean; SD: standard deviation; *T*-test, *p* > 0.05. Variable names are present in Supplementary Table S4.

Treatment	Variable Name (Unit)	M ± SD
Hand self-pollination (HSP)	Weight (mg)	0.5 ± 0.2
	Mean size (mm)	0.827 ± 0.029
	Grs (number)	14.0 ± 1.054
	Grp (%)	93.333 ± 7.027
	Mgt (days)	4.149 ± 0.163
	Mgr (day^−1^)	0.241 ± 0.009
	Gsp (%)	24.136 ± 0.906
	Unc (bit)	0.413 ± 0.386
	Syn	0.820 ± 0.180
	Vgt (day^2^)	0.246 ± 0.294
	Sdg (days)	0.380 ± 0.337
	Cvg (%)	8.928 ± 7.782
Cross-pollination (X)	Weight (mg)	0.6 ± 0.3
	Mean size (mm)	0.812 ± 0.029
	Grs (number)	12.30 ± 3.831
	Grp (%)	82.0 ± 25.541
	Mgt (days)	4.198 ± 0.306
	Mgr (day^−1^)	0.239 ± 0.015
	Gsp (%)	23.920 ± 1.543
	Unc (bit)	0.442 ± 0.429
	Syn	0.803 ± 0.197
	Vgt (day^2^)	0.635 ± 1.552
	Sdg (days)	0.460 ± 0.686
	Cvg (%)	10.057 ± 13.870

## Data Availability

The original data presented in the study are openly available in the Zenodo online repository at https://doi.org/10.5281/zenodo.15346911.

## Supplementary Materials

The following supporting information can be downloaded at https://www.mdpi.com/article/10.3390/plants14121774/s1. Table S1: Islands: populations and codes used for sampling in this study. Table S2: Morphometric and pollen characters measured in 121 female flowers and 58 male flowers throughout fourteen populations of *A. vidalii* across the nine islands of the Azores archipelago. Table S3: Different reproductive indices used to make inferences regarding the breeding strategy of *Azorina vidalii* and their classifications. Table S4: Germination variables calculated by the GerminaR package from the R environment (adapted from Lozano-Isla et al.), with limits in accordance with Ranal and Santana. Table S5: Mean and standard deviations of 23 morphometric traits applied to 121 female and 58 male flowers and 100 buds from fourteen populations of *A. vidalii*. Table S6: Scores of the six-component matrix calculated, following a Principal Component Analysis, applied to fourteen morphological floral traits, with communalities > 0.8. Table S7: Structure matrix with six canonical functions, calculated in the Discriminant analysis.

## References

[1] Tandon R., Koul M., Shivanna K.R., Tandon R., Shivanna K.R., Koul M. (2020). Reproductive Ecology of Flowering Plants: An Introduction. Reproductive Ecology of Flowering Plants: Patterns and Processes.

[2] Barrett S.C. (2010). Darwin’s legacy: The forms, function and sexual diversity of flowers. Philos. Trans. R. Soc. Lond..

[3] Sapir Y., Brunet J., Byers D.L., Imbert E., Schönenberger J., Staedler Y. (2019). Floral evolution: Breeding systems, pollinators, and beyond. Int. J. Plant Sci..

[4] Crawford D.J., Moura M., Borges Silva L., Mort M.E., Kerbs B., Schaefer H., Kelly J.K. (2019). The transition to selfing in Azorean *Tolpis* (Asteraceae). Plant Syst. Evol..

[5] Stebbins G.L. (1957). Self-fertilization and population variability in the higher plants. Am. Nat..

[6] Wright S.I., Kalisz S., Slotte T. (2013). Evolutionary consequences of self-fertilization in plants. Proc. R. Soc. B.

[7] Bazzicalupo M., Masullo I., Duffy K.J., Fay M.F., Calevo J. (2025). Seed quality and germination performance increase with cross-pollination in members of subtribe Orchidinae (Orchidaceae). Bot. J. Linn. Soc..

[8] Subaşı Ü., Güvensen A. (2025). Floral biology, pollination and reproductive success of *Campanula tomentosa* Lam. in west Anatolia. Biol. Divers. Conserv..

[9] Brys R., Jacquemyn H., Endels P., Van Rossum F., Hermy M., Triest L., De Bruyn L., Blust G.D.E. (2004). Reduced reproductive success in small populations of the self-incompatible *Primula vulgaris*. J. Ecol..

[10] Steffan-Dewenter I., Tscharntke T. (1999). Effects of habitat isolation on pollinator communities and seed set. Oecologia.

[11] POWO Plants of the World Online (2025). Facilitated by the Royal Botanic Gardens, Kew. https://powo.science.kew.org/.

[12] Nyman Y. (1993). The pollen-collecting hairs of *Campanula* (Campanulaceae). II. Function and adaptive significance in relation to pollination. Am. J. Bot..

[13] Galloway L.F., Cirigliano T., Gremski K. (2002). The contribution of display size and dichogamy to potential geitonogamy in *Campanula americana*. Int. J. Plant Sci..

[14] Nyman Y. (1993). The pollen-collecting hairs of *Campanula* (Campanulaceae). I. Morphological variation and the retractive mechanism. Am. J. Bot..

[15] D’Antraccoli M., Roma-Marzio F., Benelli G., Canale A., Peruzzi L. (2019). Dynamics of secondary pollen presentation in *Campanula medium* (Campanulaceae). J. Plant Res..

[16] Good-Avila S.V., Frey F., Stephenson A.G. (2001). The effect of partial self-incompatibility on the breeding system of *Campanula rapunculoides* L. (Campanulaceae) under conditions of natural pollination. Int. J. Plant Sci..

[17] Inoue K., Amano M. (1986). Evolution of *Campanula punctata* Lam. in the Izu Islands: Changes of pollinators and evolution of breeding systems. Plant Species Biol..

[18] Kruszewski L.J., Galloway L.F. (2006). Explaining outcrossing rate in *Campanulastrum americanum* (Campanulaceae): Geitonogamy and cryptic self-incompatibility. Int. J. Plant Sci..

[19] Shetler S.G. (1979). Pollen-collecting hairs of *Campanula* (Campanulaceae), I: Historical review. Taxon.

[20] Richardson T.E., Stephenson A.G. (1989). Pollen removal and pollen deposition affect the duration of the staminate and pistillate phases in *Campanula rapunculoides*. Am. J. Bot..

[21] Leins P., Erbar C. (1990). On the mechanisms of secondary pollen presentation in the Campanulales-Asterales-complex. Bot. Acta.

[22] Harder L.D., Johnson S.D. (2009). Darwin’s beautiful contrivances: Evolutionary and functional evidence for floral adaptation. New Phytol..

[23] Lau J.A., Galloway L.F. (2004). Effects of low-efficiency pollinators on plant fitness and floral trait evolution in *Campanula americana* (Campanulaceae). Oecologia.

[24] Olesen J.M., Alarcón M., Ehlers B.K., Aldasoro J.J., Roquet C. (2012). Pollination, biogeography and phylogeny of oceanic island bellflowers (Campanulaceae). Perspect. Plant Ecol. Evol. Syst..

[25] Milet-Pinheiro P., Santos P.S.C., Prieto-Benítez S., Ayasse M., Dötterl S. (2021). Differential evolutionary history in visual and olfactory floral cues of the bee-pollinated genus *Campanula* (Campanulaceae). Plants.

[26] Franks S.J. (2009). Genetics, evolution, and conservation of island plants. J. Plant Biol..

[27] Stuessy T.F., Takayama K., López-Sepúlveda P., Crawford D.J. (2014). Interpretation of patterns of genetic variation in endemic plant species of oceanic islands. Bot. J. Linn. Soc..

[28] Ronse De Craene L. (2018). Understanding the role of floral development in the evolution of angiosperm flowers: Clarifications from a historical and physico-dynamic perspective. J. Plant Res..

[29] Weber U.K., Nuismer S.L., Espíndola A. (2020). Patterns of floral morphology in relation to climate and floral visitors. Ann. Bot..

[30] Savriama Y. (2018). A Step-by-Step Guide for Geometric Morphometrics of Floral Symmetry. Front. Plant Sci..

[31] Gardère M.L., Florence J., Muller S., Savriama Y., Dubuisson J.Y. (2021). Codonographia Gorgonum, or the Description of a Pleiad of Bellflowers (*Campanula*, Campanulaceae) from the Cabo Verde Archipelago. Candollea.

[32] Watson H.C. (1844). Hooker’s Icones Plantarum 7 t. 684. Walp. Repert. Bot..

[33] Menezes T., Romeiras M.M., de Sequeira M.M., Moura M. (2018). Phylogenetic Relationships and Phylogeography of Relevant Lineages within the Complex Campanulaceae Family in Macaronesia. Ecol. Evol..

[34] Rego R.M.C., Moura M., Olangua-Corral M., Roxo G., Resendes R., Silva L. (2024). Anthropogenic disturbance has altered the habitat of two Azorean endemic coastal plants. BMC Ecol. Evol..

[35] Franco J.A. (1984). Nova Flora de Portugal (Continente e Açores), Volume II, Clethraceae—Compositae.

[36] Weissmann J.A., Schaefer H. (2018). The Importance of Generalist Pollinator Complexes for Endangered Island Endemic Plants. Arquipélago Life Mar. Sci..

[37] Rego R.M.C., Roxo G., Febles R., Olangua-Corral M., Fernández-Palacios O., Silva L., Moura M. Breeding Systems of Azorean Endemic Species: An Overview. Proceedings of the International Symposium FloraMac 2022.

[38] Inoue K. (1990). Evolution of Mating Systems in Island Populations of *Campanula microdonta*: Pollinator Availability Hypothesis. Plant Species Biol..

[39] Faegri K., van der Pijl L. (1979). The Principles of Pollination Ecology.

[40] Menezes T. (2013). Germinação e Desenvolvimento de *Azorina vidalii* (HC Watson) Feer (Campanulaceae) a partir de Sementes com Origem em Exemplares Silvestres da Ilha de São Miguel. Bachelor’s Dissertation.

[41] Maciel G.B. (2004). Conservação de Espécies Vasculares Endémicas dos Açores: Ecofisiologia da Germinação de Sementes de Alguns Taxa e Identificação e Caracterização de Microssatélites de *Rubus hochstetterorum* Seub. Ph.D. Dissertation.

[42] QGIS Development Team (2025). QGIS Geographic Information System. Open Source Geospatial Foundation Project. http://qgis.osgeo.org.

[43] Vogado N.O., Camargo M.G.G., Locosselli G.M., Morellato L.P.C. (2016). Edge effects on the phenology of the guamirim, *Myrcia guianensis* (Myrtaceae), a cerrado tree, Brazil. Trop. Conserv. Sci..

[44] Escobar D.F., Silveira F.A., Morellato L.P.C. (2018). Timing of seed dispersal and seed dormancy in Brazilian savanna: Two solutions to face seasonality. Ann. Bot..

[45] Dafni A., Maués M.M. (1998). A rapid and simple procedure to determine stigma receptivity. Sex. Plant Reprod..

[46] Pérez de Paz J., Febles R., Fernández-Palacios O., Olangua-Corral M., Rivero E. (2013). Manual Para la Detección de Micro-Marcadores Morfológico-Reproductivos en la Flora Canaria.

[47] Schindelin J., Arganda-Carreras I., Frise E., Kaynig V., Longair M., Pietzsch T., Preibisch S., Rueden C., Saalfeld S., Schmid B. (2012). Fiji: An open-source platform for biological-image analysis. Nat. Methods.

[48] Cruden R.W. (1977). Pollen-Ovule Ratios: A conservative indicator of breeding systems in flowering plants. Evolution.

[49] Olangua-Corral M. (2016). El Género *Argyranthemum* Webb ex Sch. Bip. (Asteraceae Anthemidae) en Gran Canaria: Evaluación de la Biodiversidad, Biología Reproductiva y Viabilidad de sus Poblaciones Naturales. Ph.D. Dissertation.

[50] Karron J.D. (1987). A comparison of levels of genetic polymorphism and self-compatibility in geographically restricted and widespread plant congeners. Evol. Ecol..

[51] Charlesworth D., Charlesworth B. (1987). Inbreeding Depression and Its Evolutionary Consequences. Annu. Rev. Ecol. Syst..

[52] Zapata T.R., Arroyo M.T.K. (1978). Plant reproductive ecology of a secondary deciduous tropical forest in Venezuela. Biotropica.

[53] Côme D., Mazliak P. (1982). Germination. Croissance et Développement. Physiologie Végétale.

[54] Kaiser H.F. (1960). The application of electronic computers to factor analysis. Educ. Psychol. Meas..

[55] Lozano-Isla F., Benites-Alfaro O.E., Pompelli M.F. (2019). GerminaR: An R package for germination analysis with the interactive web application “GerminaQuant for R”. Seed Sci. Res..

[56] Ranal M.A., Santana D.G. (2006). How and why to measure the germination process?. Braz. J. Bot..

[57] Azevedo E.B., Rodrigues M.C., Fernandes J.F., Forjaz V.H. (2004). O Clima dos Açores. Atlas Básico dos Açores.

[58] Zheng Y., Yang Z., Xu C., Wang L., Huang H., Yang S. (2020). The Interactive Effects of Daytime High Temperature and Humidity on Growth and Endogenous Hormone Concentration of Tomato Seedlings. HortScience.

[59] Vogler D.W., Peretz S., Stephenson A.G. (1999). Floral Plasticity in an Iteroparous Plant: The Interactive Effects of Genotype, Environment, and Ontogeny in *Campanula rapunculoides* (Campanulaceae). Am. J. Bot..

[60] Niu G., Heins R.D., Cameron A., Carlson W. (2001). Temperature and Daily Light Integral Influence Plant Quality and Flower Development of *Campanula carpatica* ‘Blue Clips’, ‘Deep Blue Clips’, and *Campanula* ‘Birch Hybrid’. HortScience.

[61] Tun W., Yoon J., Jeon J.S., An G. (2021). Influence of Climate Change on Flowering Time. J. Plant Biol..

[62] Gaudinier A., Blackman B.K. (2020). Evolutionary Processes from the Perspective of Flowering Time Diversity. New Phytol..

[63] Zhu J.K. (2016). Abiotic Stress Signaling and Responses in Plants. Cell.

[64] Evanhoe L., Galloway L.F. (2002). Floral Longevity in *Campanula americana* (Campanulaceae): A Comparison of Morphological and Functional Gender Phases. Am. J. Bot..

[65] Devlin B., Stephenson A.G. (1984). Factors That Influence the Duration of the Staminate and Pistillate Phases of *Lobelia cardinalis* Flowers. Bot. Gaz..

[66] Koptur S., Dávila E.N., Gordon D.R., McPhail B.D., Murphy C.G., Slowinski J.B. (1990). The Effect of Pollen Removal on the Duration of the Staminate Phase of *Centropogon talamancensis*. Brenesia.

[67] Strzałkowska-Abramek M., Jachuła J., Wrzesień M., Bożek M., Dąbrowska A., Denisow B. (2018). Nectar Production in Several *Campanula* Species (Campanulaceae). Acta Sci. Pol. Hortorum Cultus.

[68] Gao J., Xiong Y.-Z., Huang S.-Q. (2015). Effects of Floral Sexual Investment and Dichogamy on Floral Longevity. J. Plant Ecol..

[69] Bernardello G., Anderson G.J., Stuessy T.F., Crawford D.J. (2001). A Survey of Floral Traits, Breeding Systems, Floral Visitors, Pollination Systems of the Angiosperms of the Juan Fernandez Islands (Chile). Bot. Rev..

[70] Yoder J.B., Gomez G., Carlson C.J. (2020). Zygomorphic Flowers Have Fewer Potential Pollinator Species. Biol. Lett..

[71] Krishna S., Keasar T. (2018). Morphological Complexity as a Floral Signal: From Perception by Insect Pollinators to Co-Evolutionary Implications. Int. J. Mol. Sci..

[72] Kobayashi S., Inoue K., Kato M. (1999). Mechanism of Selection Favoring a Wide Tubular Corolla in *Campanula punctata*. Evolution.

[73] Crowl A.A., Miles N.W., Visger C.J., Hansen K., Ayers T., Haberle R., Cellinese N. (2016). A Global Perspective on Campanulaceae: Biogeographic, Genomic, and Floral Evolution. Am. J. Bot..

[74] Pacini E., Hesse M. (2005). Pollenkitt–Its Composition, Forms and Functions. Flora.

[75] De Oliveira D., Gomes A., Ilharco F.A., Manteigas A.M., Pinto J., Ramalho J. (2001). Importance of Insect Pollinators for the Production in the Chestnut, *Castanea sativa*. Acta Hortic..

[76] Morgado L.N., Gonçalves-Esteves V., Resendes R., Ventura M.A.M. (2018). A Pollen Inventory of Endemic Species from the Azores Archipelago, Portugal. Palynology.

[77] Moore D., Howard F.W., Moore D., Giblin-Davis R.M., Abad R.G. (2001). Insects of Palm Flowers and Fruits. Insects on Palms.

[78] Coberly L.C., Rausher M.D. (2003). Analysis of a Chalcone Synthase Mutant in *Ipomoea purpurea* Reveals a Novel Function for Flavonoids: Amelioration of Heat Stress. Mol. Ecol..

[79] Koski M.H., Galloway L.F. (2018). Geographic Variation in Pollen Color Is Associated with Temperature Stress. New Phytol..

[80] Oschwald M. (1919). Observations sur la Biologie Florale des Campanules. Bull. Soc. Bot. Genéve.

[81] Yamada T., Kodama K., Maki M. (2014). Floral Morphology and Pollinator Fauna Characteristics of Island and Mainland Populations of *Ligustrum ovalifolium* (Oleaceae). Bot. J. Linn. Soc..

[82] Thompson S.W., Lammers T.G. (1997). Phenetic analysis of morphological variation in the *Lobelia cardinalis* complex (Campanulaceae: Lobelioideae). Syst. Bot..

[83] Schaefer H., Moura M., Belo Maciel M.G., Silva L., Rumsey F.J., Carine M.A. (2011). The Linnean Shortfall in Oceanic Island Biogeography: A Case Study in the Azores. J. Biogeogr..

[84] Nogales M., Heleno R., Traveset A., Vargas P. (2012). Evidence for Overlooked Mechanisms of Long-Distance Seed Dispersal to and between Oceanic Islands. New Phytol..

[85] Ridley H.N. (1930). The Dispersal of Plants Throughout the World.

[86] Fuster-Calvo A., Nogales M., Heleno R., Vera C., Vargas P. (2021). Sea Dispersal Potential and Colonization of the Galápagos Littoral Flora. J. Biogeogr..

[87] Good-Avila S.V., Nagel T., Vogler D.W., Stephenson A.G. (2003). Effects of Inbreeding on Male Function and Self-Fertility in the Partially Self-Incompatible Herb *Campanula rapunculoides* (Campanulaceae). Am. J. Bot..

[88] Rodríguez-Rodríguez M.C., Valido A. (2011). Consequences of Plant–Pollinator and Floral–Herbivore Interactions on the Reproductive Success of the Canary Islands Endemic *Canarina canariensis* (Campanulaceae). Am. J. Bot..

[89] Cupido C.N., Nelson L.J. (2012). Floral Functional Structure, Sexual Phases, Flower Visitors and Aspects of Breeding System in *Roella ciliata* (Campanulaceae) in a Fragmented Habitat. Plant Syst. Evol..

[90] Nyman Y. (1992). Pollination mechanisms in six *Campanula* species (Campanulaceae). Plant Syst. Evol..

[91] Kaothien-Nakayama P., Isogai A., Takayama S., Pua E., Davey M. (2009). Self-Incompatibility Systems in Flowering Plants. Plant Developmental Biology—Biotechnological Perspectives.

[92] Good-Avila S.V., Stephenson A.G. (2002). The Inheritance of Modifiers Conferring Self-Fertility in the Partially Self-Incompatible Perennial, *Campanula rapunculoides* L. (Campanulaceae). Evolution.

[93] Good-Avila S.V., Stephenson A.G. (2003). Parental Effects in a Partially Self-Incompatible Herb *Campanula rapunculoides* L. (Campanulaceae): Influence of Variation in the Strength of Self-Incompatibility on Seed Set and Progeny Performance. Am. Nat..

[94] Grossenbacher D.L., Brandvain Y., Auld J.R., Burd M., Cheptou P.O., Conner J.K., Grant A.G., Hovick S.M., Pannell J.R., Pauw A. (2017). Self-Compatibility Is Over-Represented on Islands. New Phytol..

[95] Yamamoto M., Nishimura K., Kitashiba H., Sakamoto W., Nishio T. (2019). High Temperature Causes Breakdown of S-Haplotype-Dependent Stigmatic Self-Incompatibility in Self-Incompatible *Arabidopsis thaliana*. J. Exp. Bot..

[96] Montalt R., Prósper L., Vives M.C., Navarro L., Ollitrault P., Aleza P. (2022). Breakdown of Self-Incompatibility in Citrus by Temperature Stress, Bud Pollination and Polyploidization. Agriculture.

[97] De Mesquita Rodrigues J.E. (1954). Notas sobre a Cariologia de *Cistus palhinhaii* Ingram, *C. crispus* L., *Plantago maritima* L. e *Campanula vidalii* Watson. Bol. Soc. Brot..

[98] Novikova P.Y., Kolesnikova U.K., Scott A.D. (2023). Ancestral Self-Compatibility Facilitates the Establishment of Allopolyploids in Brassicaceae. Plant Reprod..

[99] Morelatto L.P.C., Alberton B., Alvarado S.T., Borges B., Buisson E., Camargo M.G.G., Cancian L.F., Carstensen D.W., Escobar D.F.E., Leite P.T.P. (2016). Linking Plant Phenology to Conservation Biology. Biol. Conserv..

