# Supplementary material for: Floral Biology, Breeding System and Conservation Implications for the Azorean Endemic Azorina vidalii (Campanulaceae)
Source: Plants (Basel). 2025 Jun 10;14(12):1774. doi: 10.3390/plants14121774 (PMC12197116; doi:10.3390/plants14121774)
Supplement: Supplementary file 1 [file plants-14-01774-s001.zip › plants-3654894-supplementary.pdf]

Supplementary Table S1. Islands, populations and codes used for sampling in this study.

Island	Population	Code
Santa Maria	Ponta do Castelo	MAPC
Santa Maria	São Lourenço	MASL
São Miguel	Areal de Santa Bárbara	SMAB
São Miguel	Capelas	SMCA
São Miguel	Fajã do Calhau	SMFC
São Miguel	Lombo Gordo	SMLG
São Miguel	Mosteiros	SMMO
São Miguel	Ponta Delgada (garden)	SMPD
Terceira	Porto Martins	TEPM
Graciosa	Ilhéu da praia	GRIP
Pico	Baixa da Ribeirinha	PIBR
Pico	São Roque	PISR
Faial	Castelo Branco	FABR
São jorge	Fajã das Almas	SJFA
São jorge	Fajã da Caldeira de Santo Cristo	SJFS
Flores	Fajã Grande	FLFG
Corvo	Vila do Corvo	COVC

Supplementary Table S2. Morphometric and pollen characters measured in 121 female flowers and 58 male flowers, throughout fourteen populations of *A. vidalii*, across the nine islands of the Azores archipelago.

Measurements taken on flowers of Azorina vidalii (Campanulaceae)		
Calix	*Cal_L*	Calix tube basal perimeter
	Cal_Sep_L	Sepal length
	Cal_Sep_W	Sepal width
	Cal_Tub_L	Calix tube length
Corolla	*Cor_L*	Corolla length (from the base to the top of the lobe)
	Cor_Dt	Maximum diameter of flower aperture (above view)
	Cor_Est_D	Maximum diameter of the flower tube (lateral view)
	Cor_Ori_P	Perimeter of aperture (above view)
	Cor_Aber_D	Internal diameter of aperture (above view)
	Cor_Lb_L	Corolla lobe length
	Cor_Lb_W	Corolla lobe width
	Cor_Est_Pm	Corolla tube mean apical perimeter (mean of all the lobes of one flower)
	Cor_Est_Pt	Corolla tube total apical perimeter (sum of all the lobes of one flower)
	Cor_Base_P	Corolla tube basal perimeter
	Cor_Est_L	Corolla tube length
Androecium	*Fil_L*	Filament length
	Ant_L	Maximum anther length
	Ant_A	Maximum anther width
	Est_L	Stamen length
Gynoecium	*StgB_L*	Stigmatic lobe length
	Sty_L	Style length
	Ova_L	Ovary length
	Ova_D	Ovary diameter
Pollen grains	*Poll_Dp*	Polar diameter
	Poll_De	Equatorial diameter
	Poll_P/E	Ratio between polar and equatorial diameters
	Poll_Ape_Lx	Mean diameter of aperture
	Poll_Spi_Lx	Mean length of spines

Supplementary Table S3. Different reproductive indices used to make inferences regarding the breeding strategy of *Azorina vidalii* and their classifications.

**Autogamy index**[1]	$\frac{(Px - Pc)}{(Px - Ps)}$	<0.1	Obligate xenogamy
		0.11-0.5	Facultative xenogamy
		0.51-0.85	Facultative autogamy
		>0.85	Obligate autogamy
**Autogamy and self-compatibility index**[2]	s(s=1–t)	≤ 0.03	Self-Incompatible (SI)
		0.03 < S ≤ 0.30	Slightly Self-Compatible (S-SC)
		>0.30	Self-Compatible (SC)
**Index of Self-Incompatibility (ISI)**[3]	$\frac{Ss}{Sx}$	0	Self-Incompatible (SI)
		<0.2	Mostly Self-Incompatible (M-SI)
		0.2>ISI<1	Partially Self-Incompatible (P-SI)
		>1	Self-Compatible (SC)
**Inbreeding depression (δ)**[4]	$1-(\frac{Ws}{Wx})$	δ>0	Inbreeding depression
		δ<0	Outcrossing depression

[1]Autogamy index, by Charlesworth and Charlesworth (1987) estimates the rate of autogamy by comparing seed production of naturally pollinated flowers (Pc), hand pollinated (Ps) and cross-pollinated ones (Px); [2]Autogamy and self-compatibility index, by Karron (1987) assumes that the rates of autogamy (s) and xenogamy (t) equal one; [3]The Index of Self-Incompatibility, from Zapata and Arroyo (1978), uses the proportion between number of seeds from hand self-pollination (Ss) and those from cross-pollination (Sx); [4]Inbreeding depression from Charlesworth and Charlesworth (1987) was calculated by comparing the seed sets of hand pollinated flowers (wg) and cross-pollinated flowers (wx).

Supplementary Table S4. Germination variables calculated by GerminaR package from R environment (adapted from Lozano-Isla et al.), with limits in accordance with Ranal and Santana.

**Variables**	**Abbreviations**	**Limits**	**Units**
Germinated seed number	Grs	0 ≤ GSN ≤ n	count
Germinability	Grp	0 ≤ GNP ≤ 100	%
Mean germination time	Mgt	0 ≤ MGT ≤ k	Time
Germination speed	Gsp	0 < GSP ≤ 100	%
Mean germination rate	Mgr	0 < MGR ≤ 1	$time^{-1}$
Synchronization index	Syn	0 ≤ SYN ≤ 1	-
Uncertainty index	Unc	0 ≤ UNC ≤ log2n	Bit
Germination standatd deviation	Sdg	0 < SDG ≤ ∞	Time
Germination variance	Vgt	0 < VGT ≤ ∞	$Time^2$
Coefficient of variation	Cvg	0 < CVG ≤ ∞	%

Supplementary Table S5. Mean and standard deviations of 23 morphometric traits applied to 121 female and 58 male flowers, and 100 buds, from fourteen populations of *A. vidalii.*

**Pop**	**Cal_L**	**Cal-Tub_L**	**Cal-Sep_W**	**Cal-Sep_L**	**Cor_L**	**Cor-Lb_L**	**Cor-Base_P**
**MAPC**	1,302±0,207	0,552±0,127	0,488±0,059	0,750±0,110	2,493±0,198	0,842±0,112	4,173±0,211
**MASL**	1,436±0,108	0,653±0,101	0,71±0,083	0,783±0,059	2,577±0,132	0,977±0,083	4,048±0,367
**SMAB**	1,334±0,066	0,666±0,077	0,788±0,059	0,668±0,038	2,722±0,096	1,138±0,100	5,272±0,175
**SMCA**	1,406±0,241	0,662±0,095	0,695±0,074	0,743±0,248	2,740±0,121	0,797±0,098	4,350±0,610
**SMFC**	1,529±0,152	0,711±0,099	0,608±0,077	0,818±0,118	3,145±0,198	0,776±0,130	4,748±0,340
**TEPM**	1,136±0,086	0,557±0,073	0,635±0,111	0,579±0,044	2,497±0,291	0,836±0,136	4,534±0,467
**GRIP**	1,191±0,108	0,565±0,070	0,656±0,159	0,626±0,120	2,868±0,236	1,080±0,096	4,583±0,306
**SJFA**	1,423±0,137	0,481±0,083	0,528±0,034	0,942±0,108	2,907±0,225	0,754±0,031	4,633±0,511
**SJFS**	1,109±0,196	0,526±0,114	0,462±0,079	0,583±0,114	2,707±0,284	0,837±0,168	3,777±0,373
**PIBR**	1,139±0,093	0,443±0,096	0,435±0,096	0,696±0,061	2,672±0,179	0,641±0,064	4,953±0,280
**PILX**	1,261±0,162	0,693±0,067	0,736±0,073	0,568±0,188	2,832±0,084	0,963±0,094	4,578±0,297
**FABR**	1,020±0,103	0,593±0,075	0,702±0,095	0,428±0,146	2,907±0,096	0,883±0,100	4,749±0,319
**FLFG**	1,140±0,143	0,497±0,117	0,556±0,124	0,644±0,123	2,911±0,312	0,972±0,085	4,269±0,864
**COVC**	0,872±0,064	0,562±0,055	0,689±0,090	0,310±0,072	2,496±0,117	1,010±0,104	5,108±0,561

**Pop**	**Cor-Est_L**	**Cor-Est_Pt**	**Cor-Est_Pm**	**Cor_Dt**	**Cor-Est_D**	**Cor-Aber_D**	**Cor-Ori_P**	**Cor-Lb_W**
**MAPC**	1,651±0,133	4,436±0,316	0,857±0,109	2,869±0,337	1,591±0,107	1,314±0,123	3,866±0,264	0,981±0,122
**MASL**	1,599±0,093	4,602±0,43	0,92±0,086	2,571±0,352	1,498±0,094	1,282±0,21	3,492±0,328	1,111±0,112
**SMAB**	1,584±0,119	4,716±0,197	0,943±0,039	2,670±0,161	1,744±0,072	1,373±0,149	4,041±0,215	1,139±0,103

**SMCA**	1,943±0,123	4,894±0,363	1,006±0,095	2,926±0,149	1,659±0,125	1,373±0,085	4,074±0,133	0,946±0,093
**SMFC**	2,369±0,124	4,099±0,378	0,820±0,076	2,832±0,133	1,61±0,108	1,207±0,034	4,202±0,773	0,946±0,082
**TEPM**	1,661±0,184	3,602±0,371	0,720±0,074	1,913±0,384	1,368±0,155	1,073±0,100	3,212±0,205	0,863±0,149
**GRIP**	1,788±0,202	4,822±0,359	0,964±0,072	3,491±0,490	1,672±0,147	1,468±0,157	4,461±0,559	1,164±0,098
**SJFA**	2,153±0,221	4,034±0,606	0,833±0,099	3,184±0,892	1,620±0,075	1,522±0,236	4,586±1,101	0,894±0,077
**SJFS**	1,870±0,176	3,671±0,464	0,734±0,093	2,249±0,293	1,333±0,133	1,093±0,075	3,236±0,304	0,827±0,134
**PIBR**	2,031±0,162	4,279±0,255	0,815±0,062	3,15±0,626	1,722±0,099	1,614±0,284	5,14±1,046	0,869±0,071
**PILX**	1,869±0,067	3,470±0,179	0,694±0,036	2,015±0,143	1,470±0,102	1,125±0,090	3,292±0,191	0,887±0,071
**FABR**	2,024±0,096	4,284±0,275	0,857±0,055	2,319±0,256	1,535±0,095	1,166±0,100	3,551±0,240	0,990±0,049
**FLFG**	1,939±0,287	4,628±0,305	0,940±0,080	2,470±0,405	1,464±0,105	1,33±0,137	3,797±0,376	1,080±0,082
**COVC**	1,486±0,108	4,772±0,497	0,935±0,091	2,321±0,206	1,535±0,125	1,289±0,181	3,698±0,479	1,132±0,100

**Pop**	**Ant_L**	**Ant_A**	**Fil_L (buds)**	**Est_L**	**Fil_L (open flowers)**	**Sty_L**	**StgB_L**	**Ova_L**
**MAPC**	0,998±0,052	0,260±0,029	0,565±0,067	1,412±0,077	0,557±0,027	1,001±0,080	0,381±0,049	0,597±0,130
**MASL**	0,981±0,015	0,277±0,009	0,696±0,027	1,476±0,037	0,659±0,033	1,152±0,097	0,337±0,033	0,554±0,064
**SMAB**	1,083±0,068	0,344±0,020	0,676±0,156	1,555±0,226	0,773±0,057	1,469±0,109	0,366±0,032	0,745±0,134
**SMCA**	1,173±0,073	0,394±0,052	0,718±0,074	1,014±0,085	0,390±0,081	1,196±0,109	0,346±0,041	0,610±0,113
**SMFC**	1,060±0,042	0,303±0,011	0,785±0,051	1,672±0,043	0,726±0,050	1,378±0,138	0,441±0,051	0,715±0,028
**TEPM**	0,942±0,089	0,312±0,016	0,688±0,071	1,444±0,077	0,672±0,059	1,059±0,079	0,378±0,048	0,581±0,060
**GRIP**	0,984±0,042	0,319±0,015	0,561±0,065	1,395±0,113	0,620±0,043	1,355±0,158	0,429±0,040	0,673±0,056
**SJFA**	1,047±0,046	0,259±0,011	0,740±0,152	1,650±0,146	0,774±0,102	1,631±0,258	0,492±0,057	0,611±0,137
**SJFS**	0,825±0,042	0,221±0,021	0,552±0,122	1,251±0,114	0,761±0,208	1,393±0,209	0,404±0,109	0,661±0,099

**PIBR**	1,014±0,072	0,294±0,022	0,675±0,122	1,497±0,161	0,758±0,051	1,366±0,118	0,435±0,043	0,683±0,077
**PILX**	0,991±0,011	0,288±0,010	0,944±0,043	1,731±0,052	0,918±0,060	1,219±0,105	0,350±0,018	0,645±0,131
**FABR**	0,921±0,040	0,294±0,018	0,629±0,148	1,411±0,190	0,727±0,044	1,336±0,085	0,364±0,064	0,608±0,047
**FLFG**	0,758±0,084	0,221±0,018	0,360±0,216	0,988±0,309	0,723±0,096	1,426±0,105	0,394±0,105	0,507±0,095
**COVC**	0,861±0,049	0,276±0,033	0,731±0,108	1,457±0,120	0,765±0,059	1,299±0,055	0,396±0,047	0,494±0,060

Supplementary Table S6. Scores of the six-component matrix calculated, following a Principal Component Analysis, applied to fourteen morphological floral traits, with communalities >0.8.

**Component Matrix**						
	**Components**					
	**1**	**2**	**3**	**4**	**5**	**6**
**Cal_L**	,481	,530	,187	,255	-,571	,055
**Cal-Sep_L**	,429	,557	,196	,095	-,619	-,129
**Cor_L**	,229	-,076	,865	,057	,149	,389
**Cor-Lb_L**	,151	-,709	,050	,448	-,208	,226
**Cor-Est_L**	,133	,346	,813	-,210	,268	,245
**Cor-Est_Pt**	,818	-,453	-,110	-,115	,031	,017
**Cor-Est_Pm**	,824	-,408	-,075	-,124	-,028	,058
**Cor_Dt**	,758	,056	,145	-,029	,196	-,449
**Cor-Ori_P**	,670	,086	,249	-,034	,308	-,481
**Cor-Lb_W**	,532	-,700	,024	,266	-,133	,028
**Ant_L**	,514	,593	-,340	,342	,249	,201
**Ant_A**	,523	,322	-,444	,222	,291	,452
**Est_L**	-,183	,205	-,034	,821	,282	-,217
**Fil_L**	-,473	-,271	,477	,546	,029	-,168
**Extraction method: Principal Component Analysis. Six components extracted.**						

Supplementary Table S7. Structure matrix with six canonical functions, calculated in the Discriminant analysis.

	Function					
	**1**	**2**	**3**	**4**	**5**	**6**
**REGR factor score 3 for analysis 5**	,872*	,127	-,362	-,047	-,246	-,171
**REGR factor score 4 for analysis 5**	,222	-,016	,825*	-,247	,175	,422
**REGR factor score 5 for analysis 5**	,091	-,086	,096	,932*	,235	,226
**REGR factor score 2 for analysis 5**	,028	,206	,056	-,029	,748*	-,627
**REGR factor score 6 for analysis 5**	,063	-,459	-,329	-,240	,625*	,479
**REGR factor score 1 for analysis 5**	-,046	,562	-,219	-,040	,224	,763*

Between-group correlations in the set between discriminant variables and standardized canonical discriminant functions
Variables ordered by absolute size of correlation in the function.
***Largest absolute correlation between each variable and any discriminant function.**
